# State Transition for Statistical SLAM Using Planar Features in 3D Point Clouds

**DOI:** 10.3390/s19071614

**Published:** 2019-04-03

**Authors:** Amirali Khodadadian Gostar, Chunyun Fu, Weiqin Chuah, Mohammed Imran Hossain, Ruwan Tennakoon, Alireza Bab-Hadiashar, Reza Hoseinnezhad

**Affiliations:** 1 School of Engineering, RMIT University, Melbourne VIC 3001, Australia; amirali.khodadadian@rmit.edu.au (A.K.G.); s3538114@student.rmit.edu.au (W.C.); s3638951@student.rmit.edu.au (M.I.H.); ruwan.tennakoon@rmit.edu.au (R.T.); abh@rmit.edu.au (A.B.-H.); rezah@rmit.edu.au (R.H.); 2 State Key Laboratory of Mechanical Transmissions, School of Automotive Engineering, Chongqing University, Chongqing 400044, China

**Keywords:** simultaneous localization and mapping, planar features, plane parameters, transition model, Bayesian filters

## Abstract

There is a large body of literature on solving the SLAM problem for various autonomous vehicle applications. A substantial part of the solutions is formulated based on using statistical (mainly Bayesian) filters such as Kalman filter and its extended version. In such solutions, the measurements are commonly some point features or detections collected by the sensor(s) on board the autonomous vehicle. With the increasing utilization of scanners with common autonomous cars, and availability of 3D point clouds in real-time and at fast rates, it is now possible to use more sophisticated features extracted from the point clouds for filtering. This paper presents the idea of using planar features with multi-object Bayesian filters for SLAM. With Bayesian filters, the first step is prediction, where the object states are propagated to the next time based on a stochastic transition model. We first present how such a transition model can be developed, and then propose a solution for state prediction. In the simulation studies, using a dataset of measurements acquired from real vehicle sensors, we apply the proposed model to predict the next planar features and vehicle states. The results show reasonable accuracy and efficiency for statistical filtering-based SLAM applications.

## 1. Introduction

Simultaneous Localization and Mapping (SLAM) is often considered as one of the main challenges in the field of robotics and autonomous vehicles [1,2]. The aim of SLAM is to build a map of an unknown environment while simultaneously determining the location of the vehicle within this map. Neither the map nor the vehicle position are known in advance. However, a kinematic model of the vehicle motion is assumed to be known a priori, and the unknown environment is populated with artificial or natural landmarks.

There is a large body of literature on solving the SLAM problem for various autonomous vehicle applications. Many of the solutions are formulated based on using a Bayesian filter that recursively propagates the distribution of the vehicle’s dynamic states (and sometimes the map features). Davison [3] proposed a solution based on using Extended Kalman Filters (EKFs) to track the location of each map feature which was extracted from the sensor measurements. To improve the accuracy of this method, Thrun et al. [4] proposed the Sparse Extended Information Filter. The idea is to optimize the calculations required by the EKF, via taking advantage of sparse matrices. Montemerlo et al. [5] proposed a Rao-Blackwellised particle filter-based method called FastSLAM. In this method, the odometry uncertainties are rectified by adding random offsets to the odometry data for each particle.

In such solutions, to simultaneously estimate both vehicle and landmark locations, the Bayesian filter needs to employ two models: an observation model, and a motion model. With regards to the observations, the vehicle must be equipped with a sensor or set of sensors that produce measurements related to the surrounding landmark locations. The most common examples are LIDAR [6,7,8], RGB camera [9,10,11,12], RGB-D camera [13,14] and sonar [15] sensors. The sensory measurements usually have range limitations, and contain measurement noise. The raw measurements are normally processed further to extract point features. An on-board sensory system on the vehicle is usually in place to collect the features in real-time.

With the advancement of sensor technology and decreasing cost of LIDAR scanners, the generation of an adequate 3D point cloud is now possible. A LIDAR works by sending a laser pulse towards an object and measuring the time it takes to reflect from the object and return to the sensor. Because of its high accuracy and ease of use, a LIDAR sensor has become a common choice for SLAM purposes. Having the generated point cloud, it is possible to extract features that are more complex (and convey more information) than point features. Employing more information-rich features as observations in a Bayesian filter within a SLAM algorithm can increase the algorithm efficiency and reduce the computational cost. For example employing planar features instead of 3D points could considerably enhance the algorithm efficiency, as the number of planes is significantly smaller than the number of points in a typical 3D point cloud. However, in order to properly use these features in the Bayesian framework, an appropriate motion model needs to be devised.

In human-inhabited environments, buildings and large facilities are currently almost ubiquitous and their geometric profiles comprise a large number of plane shapes. Out of all these shapes, the major ones naturally create “obstacles” or define “edges” beyond which the vehicles cannot protrude, and these planes can be extracted and employed as features or landmarks in SLAM applications. Indeed, due to the geometric simplicity of the plane shapes and their abundance in human-inhabited environments, planar features have attracted increasing attention from both the computer graphics and robotics community in recent years [16,17,18]. As for the smaller plane shapes, they may be from the profiles of small objects or even moving objects such as vehicles. These small planes can be readily excluded from the set of landmarks by applying a threshold to the dimension of planes.

This paper presents for the first time the idea of using planar features extracted from a 3D point cloud, instead of point features, for Bayesian SLAM filters. The major contribution of this study lies in proposing a novel transition model that predicts the parameters of planar features extracted from a 3D point cloud. With Bayesian filters, the first step is prediction, where the object state densities are propagated to the next time step based on a stochastic transition model (motion model). We present how such a transition model can be developed, and propose a solution for state prediction. In the simulation studies, using a dataset of measurements acquired from real vehicle sensors, we apply the model to predict the planar features at the next time. The results show reasonable accuracy and efficiency for statistical filtering-based SLAM applications.

The proposed transition model consists of two sub-models: the plane transition model and the vehicle transition model. The latter one is associated with localization; namely, it predicts the vehicle states at time k+1 using the state information at time *k*. The former one is associated with mapping, which predicts the feature parameters at time k+1 based on the feature information at time *k* as well as the predicted vehicle states resulting from the latter model. The accuracy and effectiveness of the proposed transition model are verified using real-world point cloud measurements from the KITTI dataset [19]. The graphical and numerical simulation results show that the predicted planar features resulting from the proposed transition model closely match the measured features (i.e., segmented 3D planes) at time k+1, in terms of the plane dimensions (plane area and vertex distance) and the plane orientation (normal vector).

The rest of the paper is organized as follows. Section 2 provides relevant theoretical background. Section 3 introduces the details of the proposed transition model which comprises two sub-models: the plane transition model and the vehicle transition model. Section 4 explains the detailed procedure for implementation of the proposed transition model. Section 5 provides verification of the proposed transition model by means of graphical and numerical simulation results. Section 6 concludes the paper.

## 2. Background

The proposed transition model is mainly designed to be used with a recently developed Random Finite Set (RFS) based Bayesian filter—the Labeled Multi-Bernoulli (LMB) filter [20]. In this section, we provide the theoretical background necessary for understanding this filter, followed by a brief introduction to the KITTI dataset [19] which was used for simulation studies in this work.

### 2.1. Labeled Multi-Bernoulli Filter

In this section, we introduce the Labeled Multi-Bernoulli (LMB) filter [20]. We adopt the same notation used in [20] where the single-object states are denoted by lower-case letters, e.g., *x*, x and multi-object states by upper-case letters, e.g., *X*, X. In order to distinguish between labeled and unlabeled states and their distributions, the labeled one is shown by bolded letters, e.g., x, X, etc., spaces by blackboard bold, e.g., X, L, C, etc., and the class of finite subsets of a space X by F(X). Following [20], throughout the paper, the standard inner product notation is used and denoted by 〈f,g〉≜∫f(x)g(x)dx, the generalized Kronecker delta is denoted by

$$\delta_{Y}\left(X\right)≜\left\{\begin{array}{c} 1,\;\mathrm{if}\;X=Y\\ 0,\;\mathrm{otherwise} \end{array}\right.,$$

and the inclusion function, a generalization of the indicator function, by

$$1_{Y}\left(X\right)≜\left\{\begin{array}{c} 1,\;\mathrm{if}\;X\subseteq Y\\ 0,\;\mathrm{otherwise} \end{array}\right..$$

The LMB RFS is completely described by its components $\pi =\{\left(r^{\left(\ell \right)},p^{\left(\ell \right)}\right):\ell \in L\}$. The LMB RFS density is given by $\pi \left(X\right)=\Delta \left(X\right)w\left(L\left(X\right)\right){\left[p\right]}^{X},$ where $p\left(x,\ell \right)=p^{\left(\ell \right)}\left(x\right)$ and $w\left(L\right)=\prod_{i\in L}\left(1-r^{\left(i\right)}\right)\prod_{\ell \in L}\frac{1_{L}\left(\ell \right)r^{\left(\ell \right)}}{\left(1-r^{\left(\ell \right)}\right)}$ comprising a single component [20]. The LMB multi-target Bayes recursion propagates multi-target posterior density at each time step according to the Chapman–Kolmogorov and the Bayes rule.

#### 2.1.1. Prediction

Assume that the prior and birth labeled multi-Bernoulli sets are modeled as follows:

(1)π(X)=Δ(X)w(L(X))pX(2)πB(X)=Δ(X)wB(L(X))pBX

where

(3)w(L)=∏i∈L1−r(i)∏ℓ∈L1L(ℓ)r(ℓ)1−r(ℓ),(4)wB(L)=∏i∈B1−rB(i)∏ℓ∈L1B(ℓ)rB(ℓ)1−rB(ℓ),

(5)p(x,ℓ)=p(ℓ)(x)(6)pB(x,ℓ)=pB(ℓ)(x).

with state space X and label space $L_{+}=B\cup L$ and with the condition B∩L=∅. The predicted multi-object distribution is then a LMB RFS and given by

(7) $$\pi_{\;+}\left(X\right)=\Delta \left(X\right)w_{+}\left(L\left(X\right)\right){\left[p_{+}\right]}^{X}$$

where

(8)w+(I+)=wS(I+∩L)wB(I+∩B)(9)wS(L)=1−r(·)ηS(·)Lr(·)ηS(·)1−r(·)ηS(·)L,(10)ηS(ℓ)=pS(·,ℓ),p(·,ℓ)(11)p+(x,ℓ)=1L(ℓ)p+,S(x,ℓ)+1B(ℓ)pB(x,ℓ)(12)p+,S(x,ℓ)=pS(·,ℓ)f(x|·,ℓ),p(·,ℓ)ηS(ℓ)

where $p_{S}\left(\cdot \right|\ell$) is the survival probability of an object and f(x|·,ℓ) is the single-object transition model. Thus, if the multi-target posterior density is an LMB RFS with parameter set $\pi =\{\left(r^{\left(\ell \right)},p^{\left(\ell \right)}\right):\ell \in L\}$ with state space X and label space L and the birth model is also an LMB RFS with parameter set $\pi_{B}=\left\{\left(r_{B}^{\left(\ell \right)},p_{B}^{\left(\ell \right)}\right):\ell \in B\right\}$ with state space X and label space B then the predicted multi-target density is also an LMB RFS with state space X and label space $L_{+}=B\cup L\left(B\cap L=\emptyset \right)$ and it is given by

(13) $$\pi_{+}=\left\{\left(r_{+,S}^{\left(\ell \right)},p_{+,S}^{\left(\ell \right)}\right):\ell \in L\right\}\cup \left\{\left(r_{B}^{\left(\ell \right)},p_{B}^{\left(\ell \right)}\right):\ell \in B\right\}$$

where

(14)r+,S(ℓ)=ηS(ℓ)r(ℓ),(15)p+,S(ℓ)=pS(·,ℓ)f(x|·,ℓ),p(·,ℓ)ηS(ℓ),

for more details see— [20]—proposition 2.

#### 2.1.2. Update

If the predicted multi-target density is an LMB RFS with parameter set $\pi_{+}=\left\{\left(r_{+}^{\left(\ell \right)},p_{+}^{\left(\ell \right)}\right):\ell \in L_{+}\right\}$, the multi-target posterior is then given by

(16) $$\pi \left(\cdot |Z\right)=\{\left(r^{\left(\ell \right)},p^{\left(\ell \right)}\left(\cdot \right)):\ell \in L_{+}\right\}$$

where

(17)r(ℓ)=∑(I+,θ)∈F(L+)×ΘI+w(I+,θ)(Z)1I+(ℓ),(18)p(ℓ)(x)=1r(ℓ)∑(I+,θ)∈F(L+)×ΘI+w(I+,θ)(Z)1I+(ℓ)p(θ)(x,ℓ),

where $\Theta_{I_{+}}$ denotes the space of mapping $\theta \;:\;I_{+}\;\to \;\left\{0,1,\cdots ,|Z|\right\}$ and,

(19)w(I+,θ)(Z)∝w+(I+)ηZ(θ)I+(20)p(θ)(x,ℓ|Z)=p+(x,ℓ)ψZ(x,ℓ;θ)ηZ(θ)(ℓ),(21)ηZ(θ)(ℓ)=〈p+(·,ℓ),ψZ(·,ℓ;θ)〉,ψZ(x,ℓ;θ)=δ0(θ(ℓ))qD(x,ℓ)(22)+(1−δ0(θ(ℓ)))pD(x,ℓ)g(zθ(ℓ)|x,ℓ)κ(zθ(ℓ))

where, g(z|x) is the single-sensor measurement likelihood, $p_{{}_{D}}\left(\cdot ,\ell \right)$ denotes probability of detection, $q_{{}_{D}}\left(\cdot ,\ell \right)=1-p_{{}_{D}}\left(\cdot ,\ell \right)$ is the probability of a missed detection, and κ(·) intensity function of the Poisson distributed clutter process.

### 2.2. KITTI Dataset

The KITTI dataset [19] was recorded in and around Karlsruhe, Germany using a VW wagon equipped with various types of sensors including a 3D laser scanner, four video cameras and a GPS/IMU navigation system. The data was collected in both urban and suburban areas (city, rural, and highway) and information was gathered over several days. In this dataset, many of the scenes are dominated by large buildings with planar surfaces. The KITTI dataset has been widely used in mobile robotics and autonomous driving research.

## 3. Proposed Transition Model

As explained in Section 1, a transition model is required in SLAM filters to predict the states of the features at the next time step, based on the state information at the current time step. Specifically, in this study, we are interested in constructing a transition model for predicting the plane parameters at the next time step, using the current plane parameters estimated by a SLAM filter. Note that the planes involved in the transition model are expressed in the vehicle coordinate system (fixed to the vehicle), as the point cloud measurements are acquired by a laser scanner mounted on top of the experimental vehicle. Thus the plane parameters vary with time, as long as the experimental vehicle is in motion. On the other hand, if seen from the global coordinate system (fixed to the ground), the plane parameters are invariant since the planes are all static in this coordinate system.

We emphasize that the vehicle is assumed to move in a static environment. If this is not the case, we make the practical assumption that planar surfaces on other moving objects are small enough to be excluded from the set “plane observations” extracted from the 3D point cloud. That set is expected to include only relatively large planar surfaces such as walls of buildings along the road.

### 3.1. Plane Transition Model

**Figure 1:**
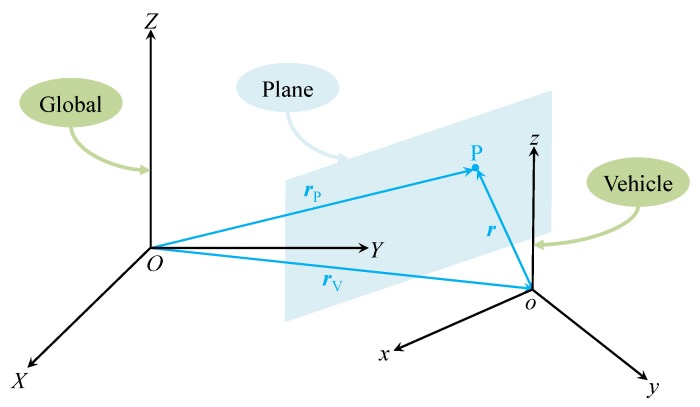
An example of the global and vehicle coordinate systems.

To facilitate the design of the transition model, the two aforementioned coordinate systems are established, as shown in Figure 1. The global coordinate system OXYZ is fixed to the ground, and the vehicle coordinate system oxyz is attached to the vehicle Center of Mass and moves along with the vehicle. P is a point on a plane segmented from the 3D point cloud obtained from the on-board laser scanner. $r_{P}$ and r are the position vectors of P in the global and vehicle coordinate systems, and $r_{V}$ is the position vector of the vehicle centre of mann (i.e., origin *o*) in the global coordinate system. The global coordinates of P are invariant since $r_{P}$ is static as seen from the global coordinate system. However, the local coordinates of P, expressed in the vehicle coordinate system, vary with time due to the motion of the vehicle. As a result, the plane parameters expressed in the vehicle coordinate system also evolve with time, and a transition model is thus needed to predict the change of plane parameters for the purpose of accurate and effective SLAM.

The plane equation, expressed in the vehicle coordinate system, is assumed to take the following form in this study:

(23) ax+by+cz+d=0,

where *x*, *y* and *z* are the coordinates of a point on the plane, and *a*, *b*, *c* and *d* are the plane parameters. Note that other forms of plane equations are also available in the literature [21], but all forms of plane equations can eventually lead to Equation (23) after simple algebraic manipulation. We may rewrite Equation (23) in the following vector form:

(24) $$\beta^{T}r=0,$$

where $\beta ={\left[a\;b\;c\;d\right]}^{T}$ and $r={\left[x\;y\;z\;1\right]}^{T}$.

**Figure 2:**
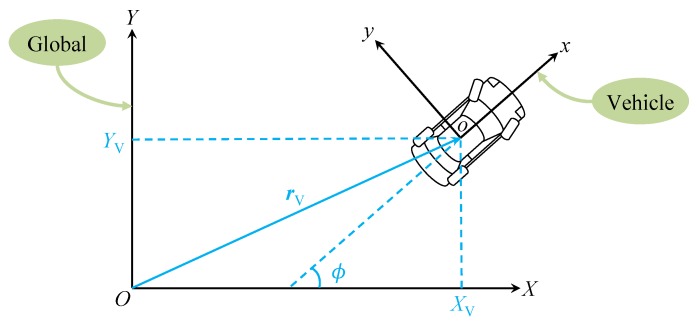
Top view of the global and vehicle coordinate systems.

Assuming that the vehicle is only performing a planar motion (which is normally the case in common flat-road urban driving scenarios), the coordinates of point P in the global coordinate system $r_{P}={\left[X\;Y\;Z\;1\right]}^{T}$, and the coordinates of point P in the vehicle coordinate system $r={\left[x\;y\;z\;1\right]}^{T}$, are related according to the following equations describing the kinematics of the vehicle [22,23]:

(25) $$r_{P}=Rr$$

and

(26) $$R=\left[\begin{array}{cccc} \cos\phi & -\sin\phi & 0 & X_{V}\\ \sin\phi & \cos\phi & 0 & Y_{V}\\ 0 & 0 & 1 & 0\\ 0 & 0 & 0 & 1 \end{array}\right],$$

where ϕ represents the heading angle of the vehicle (with respect to the *X* axis), and $X_{V}$ and $Y_{V}$ denote the coordinates of the vehicle centre of mass (origin *o*) in the global coordinate system (namely $r_{V}={\left[X_{V}\;Y_{V}\;0\;1\right]}^{T}$). The vehicle states ϕ, $X_{V}$ and $Y_{V}$ are graphically shown in Figure 2.

Equations (25) and (26) can be rewritten as follows, for time *k*:

(27) $$r_{P}=R_{k}r_{k}$$

and

(28) $$R_{k}=\left[\begin{array}{cccc} \cos\phi_{k} & -\sin\phi_{k} & 0 & X_{V,k}\\ \sin\phi_{k} & \cos\phi_{k} & 0 & Y_{V,k}\\ 0 & 0 & 1 & 0\\ 0 & 0 & 0 & 1 \end{array}\right],$$

where $r_{k}={\left[x_{k}\;y_{k}\;z_{k}\;1\right]}^{T}$. Similarly, we have the following equations for time k+1:

(29) $$r_{P}=R_{k+1}r_{k+1}$$

and

(30) $$R_{k+1}=\left[\begin{array}{cccc} \cos\phi_{k+1} & -\sin\phi_{k+1} & 0 & X_{V,k+1}\\ \sin\phi_{k+1} & \cos\phi_{k+1} & 0 & Y_{V,k+1}\\ 0 & 0 & 1 & 0\\ 0 & 0 & 0 & 1 \end{array}\right],$$

where $r_{k+1}={\left[x_{k+1}\;y_{k+1}\;z_{k+1}\;1\right]}^{T}$.

The vector $r_{P}$ is invariant with time, since it is the position vector of P in the global coordinate system. As a result, one can achieve the following equation by combining Equations (27) and (29):

(31) $$r_{k+1}=R_{k+1}^{-1}R_{k}r_{k}.$$

Note that Equation (24) can be rewritten as follows, for time *k*:

(32) $$\beta_{k}^{T}M_{k}^{-1}M_{k}r_{k}=0,$$

where $M_{k}$ represents a 4-by-4 invertible matrix. We might as well let $M_{k}=R_{k+1}^{-1}R_{k}$ and combine Equations (31) and (32), then we arrive at:

(33) $$\beta_{k}^{T}M_{k}^{-1}r_{k+1}=0.$$

The plane Equation (24) for time k+1 can be expressed as follows:

(34) $$\beta_{k+1}^{T}r_{k+1}=0.$$

Combining Equations (33) and (34) leads to the following transition model for plane parameters:

(35) $$\beta_{k+1}^{T}=\beta_{k}^{T}M_{k}^{-1},$$

where $M_{k}^{-1}={\left(R_{k+1}^{-1}R_{k}\right)}^{-1}=R_{k}^{-1}R_{k+1}$.

This plane transition model indicates that the plane parameters at time k+1 can be calculated based on the plane parameter information at time *k* and a vehicle-motion-dependent 4-by-4 matrix $M_{k}^{-1}$. Note that the computation of matrix $M_{k}^{-1}=R_{k}^{-1}R_{k+1}$ requires the inverse of $R_{k}$. It can be easily proven that the matrix $R_{k}$, expressed by Equation (28), has full rank and its inverse $R_{k}^{-1}$ exists at all times. Thus, the matrix $M_{k}^{-1}$ can be computed as long as $R_{k+1}$ is available. However, this matrix $R_{k+1}$ (as indicated by Equation (30)) requires the vehicle state information at the future time k+1, which is not available in real-time SLAM applications. To tackle this issue, in the following section we will introduce a new vehicle transition model and demonstrate how the vehicle states at time k+1 can be predicted.

### 3.2. Vehicle Transition Model

As mentioned above, the transformation matrix $R_{k+1}$ requires the unknown information from the future time k+1. In order to obtain matrix $R_{k+1}$, in this section a vehicle transition model is introduced to predict the future vehicle states at time k+1, using the available information at time *k*.

In Bayesian filtering, the Constant Turn (CT) model [24,25,26] which describes the maneuver of the vehicle motion is commonly used. This model is formulated based on the assumption that the vehicle maneuvers with a (nearly) constant speed and a (nearly) constant angular rate [24]. Based on the CT model, an Extended Constant Turn (ECT) model is proposed in this work, which can be mathematically expressed as follows:

(36) $$x_{k+1}=F_{k}x_{k}+Gu,$$

with

$$x_{k+1}={\left[X_{V,k+1}\;{\dot{X}}_{V,k+1}\;Y_{V,k+1}\;{\dot{Y}}_{V,k+1}\;\phi_{k+1}\;{\dot{\phi }}_{k+1}\right]}^{T},$$

$$x_{k}={\left[X_{V,k}\;{\dot{X}}_{V,k}\;Y_{V,k}\;{\dot{Y}}_{V,k}\;\phi_{k}\;{\dot{\phi }}_{k}\right]}^{T},$$

$$u={\left[u_{X}\;u_{Y}\;u_{\phi }\right]}^{T},$$

$$F_{k}=\left[\begin{array}{cccccc} 1 & \frac{\sin({\dot{\phi }}_{k}T)}{{\dot{\phi }}_{k}} & 0 & \frac{\cos({\dot{\phi }}_{k}T)-1}{{\dot{\phi }}_{k}} & 0 & 0\\ 0 & \cos({\dot{\phi }}_{k}T) & 0 & -\sin({\dot{\phi }}_{k}T) & 0 & 0\\ 0 & \frac{1-\cos({\dot{\phi }}_{k}T)}{{\dot{\phi }}_{k}} & 1 & \frac{\sin({\dot{\phi }}_{k}T)}{{\dot{\phi }}_{k}} & 0 & 0\\ 0 & \sin({\dot{\phi }}_{k}T) & 0 & \cos({\dot{\phi }}_{k}T) & 0 & 0\\ 0 & 0 & 0 & 0 & 1 & T\\ 0 & 0 & 0 & 0 & 0 & 1 \end{array}\right]\;,\;\mathrm{and}\;G=\left[\begin{array}{ccc} \frac{T^{2}}{2} & 0 & 0\\ T & 0 & 0\\ 0 & \frac{T^{2}}{2} & 0\\ 0 & T & 0\\ 0 & 0 & \frac{T^{2}}{2}\\ 0 & 0 & T \end{array}\right]\;,$$

where $X_{V,k}$, $Y_{V,k}$, $X_{V,k+1}$ and $Y_{V,k+1}$ denote the vehicle longitudinal and lateral displacements (i.e., the coordinates of the vehicle centre of mass as introduced in Section 3.1) in the global coordinate system at time *k* and k+1, $\phi_{k}$ and $\phi_{k+1}$ represent the vehicle heading angles at time *k* and k+1, $u_{X}$, $u_{Y}$ and $u_{\omega }$ are the vehicle velocity and yaw rate noise components, and *T* stands for the sampling period. By means of this vehicle transition model (36), the required vehicle state information at time k+1 can be predicted and in turn the matrices $R_{k+1}$ and $M_{k}^{-1}$ can be calculated.

Note that the plane transition model proposed in Section 3.1 (i.e., Equation (35)), along with the above ECT vehicle transition model, constitutes a complete state transition model that propagates the state densities of the planar features to the next time. This state transition model plays a key role in the prediction step for prospective planar-feature-based SLAM filters.

## 4. Implementation

In this section, we introduce the Sequential Monte Carlo (SMC) implementation of the proposed transition model, for the recently developed LMB filter. The LMB filter has been used in many applications such as target tracking [27,28], SLAM [29], visual tracking [30], and sensor control [31,32]. In the SMC implementation, the LMB posterior for the map features (planes) at each time step are represented by a set of particles. These particles are propagated using the proposed transition model to obtain the LMB posterior at the next time step.

Assuming the following LMB posterior $\pi_{k}$ at time *k*:

(37) $$\pi_{k}={\left\{\left(r_{k}^{\left(\ell \right)},p_{k}^{\left(\ell \right)}\right)\right\}}_{\ell \in L},$$

where *ℓ* represents the label of a plane, $r_{k}^{\left(\ell \right)}$ denotes the existence probability of the plane *ℓ*, $p_{k}^{\left(\ell \right)}$ is the spatial distribution of its parameters, and L stands for the label space. In an SMC implementation, the spatial distribution is represented by a set of weighted samples [28], namely:

(38) $$p_{k}^{\left(\ell \right)}=\sum_{i=1}^{N^{\left(\ell \right)}}\omega_{i}^{\left(\ell \right)}\delta \left(a-a_{i,k}^{\left(\ell \right)}\right)\delta \left(b-b_{i,k}^{\left(\ell \right)}\right)\delta \left(c-c_{i,k}^{\left(\ell \right)}\right)\delta \left(d-d_{i,k}^{\left(\ell \right)}\right),$$

where $N^{\left(\ell \right)}$ represents the number of particles, $\omega_{i}^{\left(\ell \right)}$ denotes the weight of the *i*-th particle, and δ is the Dirac delta function.

For each particle $\beta_{i,k}^{\left(\ell \right)}={\left[a_{i,k}^{\left(\ell \right)},b_{i,k}^{\left(\ell \right)},c_{i,k}^{\left(\ell \right)},d_{i,k}^{\left(\ell \right)}\right]}^{T}\;$ at time *k*, we are able to calculate the corresponding predicted particle $\beta_{i,k+1}^{\left(\ell \right)}={\left[a_{i,k+1}^{\left(\ell \right)},b_{i,k+1}^{\left(\ell \right)},c_{i,k+1}^{\left(\ell \right)},d_{i,k+1}^{\left(\ell \right)}\right]}^{T}$ at time k+1 based on the transition model proposed in Section 3. As a result, after the prediction stage, we will end up with a new set of particles with the same weights, namely:

$${\left\{\omega_{i,k+1}^{\left(\ell \right)},\beta_{i,k+1}^{\left(\ell \right)}\right\}}_{i=1}^{N^{\left(\ell \right)}},$$

where $\omega_{i,k+1}^{\left(\ell \right)}=\omega_{i,k}^{\left(\ell \right)}$. Then, based on the new predicted particles at time k+1, the plane parameter estimate ${\hat{\beta }}_{k+1}^{\left(\ell \right)}$ can be achieved from the distribution particles as the Expected A Posteriori (EAP) estimate (i.e., weighted average), or the Maximum A Posteriori (MAP) estimate (i.e., considering the particle with the largest weight as the estimate).

The above implementation procedure is illustrated step by step in Algorithm 1, in the form of a pseudo-code.

**Algorithm 1** Step-by-Step Implementation of the Proposed Transition Model**Require:** vehicle states $X_{V,k}$, $Y_{V,k}$, $\phi_{k}$, and particles ${\left\{\omega_{i,k}^{\left(\ell \right)},\beta_{i,k}^{\left(\ell \right)}\right\}}_{i=1}^{N^{\left(\ell \right)}}$ representing the distribution of each plane with label *ℓ*, for all labels ℓ∈L  1:  compute matrix $R_{k}$▹ use Equation (28)  2:  generate a noise sample u∼N(0,Σ)▹Σ∼ diag(noise variances)  3:  generate $X_{V,k+1}$, $Y_{V,k+1}$ and $\phi_{k+1}$▹ use Equation (36)  4:  compute matrix $R_{k+1}$▹ use Equation (30)  5:  $M_{k}^{-1}\leftarrow R_{k}^{-1}R_{k+1}$  6:  **for**
ℓ∈L
**do**  7:  **for**
$i⩽N^{\left(\ell \right)}$
**do**  8:     ${\beta_{i,k+1}^{\left(\ell \right)}}^{T}\leftarrow {\beta_{i,k}^{\left(\ell \right)}}^{T}\times M_{k}^{-1}$  9:     $\omega_{i,k+1}^{\left(\ell \right)}\leftarrow \omega_{i,k}^{\left(\ell \right)}$10:  **end for**11:  ${\hat{\beta }}_{k+1}^{\left(\ell \right)}\leftarrow \sum_{i=1}^{N^{\left(\ell \right)}}\omega_{i,k+1}^{\left(\ell \right)}\beta_{i,k+1}^{\left(\ell \right)}$▹ compute the EAP estimate12:  **end for**

## 5. Simulation Results

In this section, we demonstrate how well the proposed transition model performs, compared with the actually measured results from the KITTI dataset. Specifically, we generate the predicted planes at time k+1 using the plane information at time *k*, by means of the proposed transition model, and we segment planes from the point cloud data in the KITTI dataset at time k+1, by means of the Modified Selective Statistical Estimator (MSSE) [33]. Then, the predicted planes at time k+1 are compared with the segmented planes at time k+1, and both graphical and numerical results are demonstrated to show the closeness of the predicted planes and the segmented planes.

The point cloud data from the folder “2011_09_26_drive_0005_sync” in the KITTI dataset are employed in the simulation studies for plane segmentation. The details for accessing and using the KITTI dataset are available in [19]. The folder “2011_09_26_drive_0005_sync” includes 154 time steps (i.e., k∈[0,153]), and the points obtained at k=11, k=12, k=124 and k=125 are used in the simulation. The number of points at k=11, k=12, k=124 and k=125 are 123,334, 123,316, 118,950 and 118,572 respectively.

### 5.1. Graphical Results

**Figure 3:**
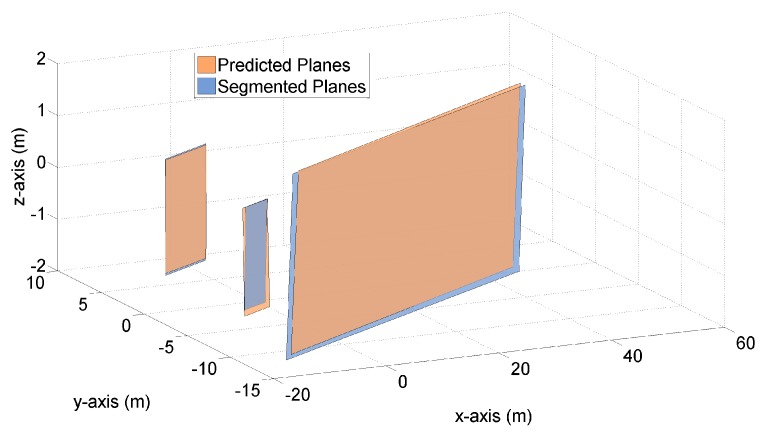
Three pairs of predicted and segmented planes at time k=125.

**Figure 4:**
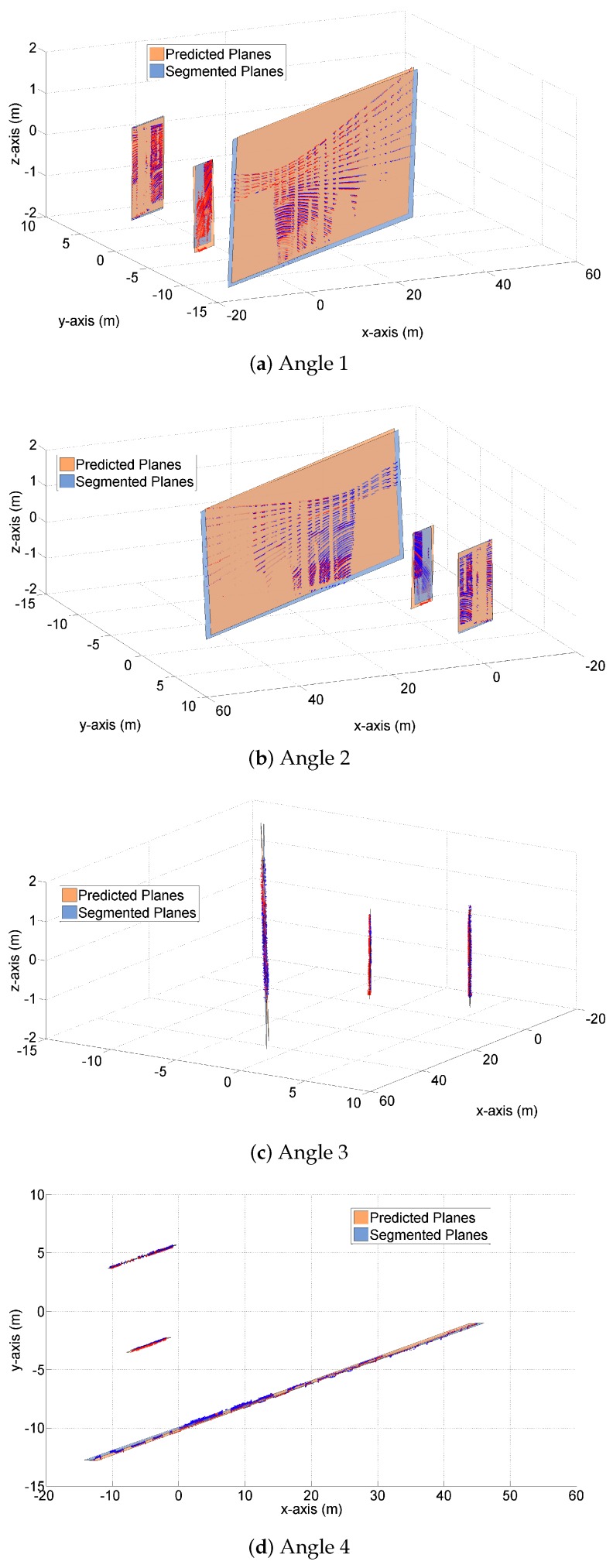
Multi-angle comparison of the predicted and segmented planes at time k=125.

Figure 3 and Figure 4 demonstrate both the predicted planes and the segmented planes at time k=125 for one Monte Carlo (MC) run. The predicted planes are in orange color, and are generated by the proposed transition model using the LIDAR measurements and vehicle states at time k=124. The segmented planes are in blue color, and are plotted based on the point cloud measurements at time k=125. Apart from the planes themselves, the predicted and measured point clouds at time k=125 are also plotted in Figure 4, on top of the corresponding planes.

Figure 3 shows that by means of the transition model and the available information at time k=124, three planes are predicted to exist at time k=125. Also, three segmented planes are present in this figure based on the LIDAR measurements at time k=125. It is clearly observed in Figure 3 that each predicted plane is associated with one segmented plane. Namely, these six planes constitute three pairs of planes, with each pair composed of one predicted plane and one segmented plane. Besides, we see that for each pair of orange and blue planes, the areas of planes are fairly close and the distances between each pair of plane vertexes are rather small (‘Each pair of plane vertexes’ is referred to one vertex on the predicted plane and its closest counterpart on the segmented plane).

Figure 4 demonstrates the three pairs of planes from different angles. Again, we observe the closeness of the predicted and segmented planes in terms of plane areas and vertex distances, when viewed from different angles. Figure 4c shows a ‘side view’ of the three pairs of planes, from which we clearly see that the predicted and segmented planes (and their normal vectors) almost coincide. Similar results are also observed in Figure 4d where a ‘top view’ of the planes is shown.

**Figure 5:**
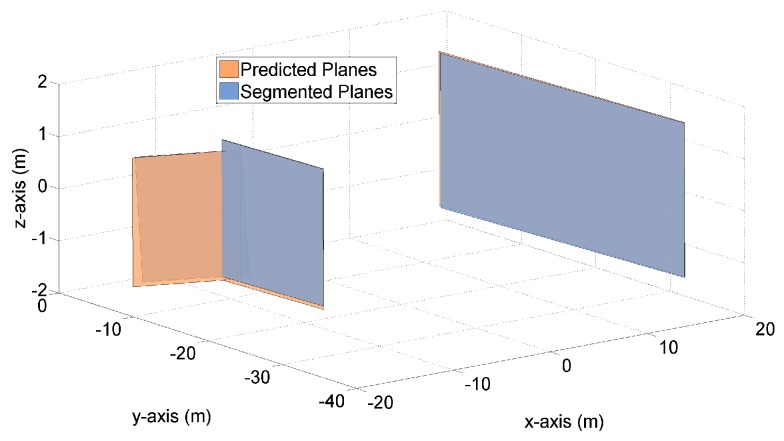
Three pairs of predicted and segmented planes at time k=12.

**Figure 6:**
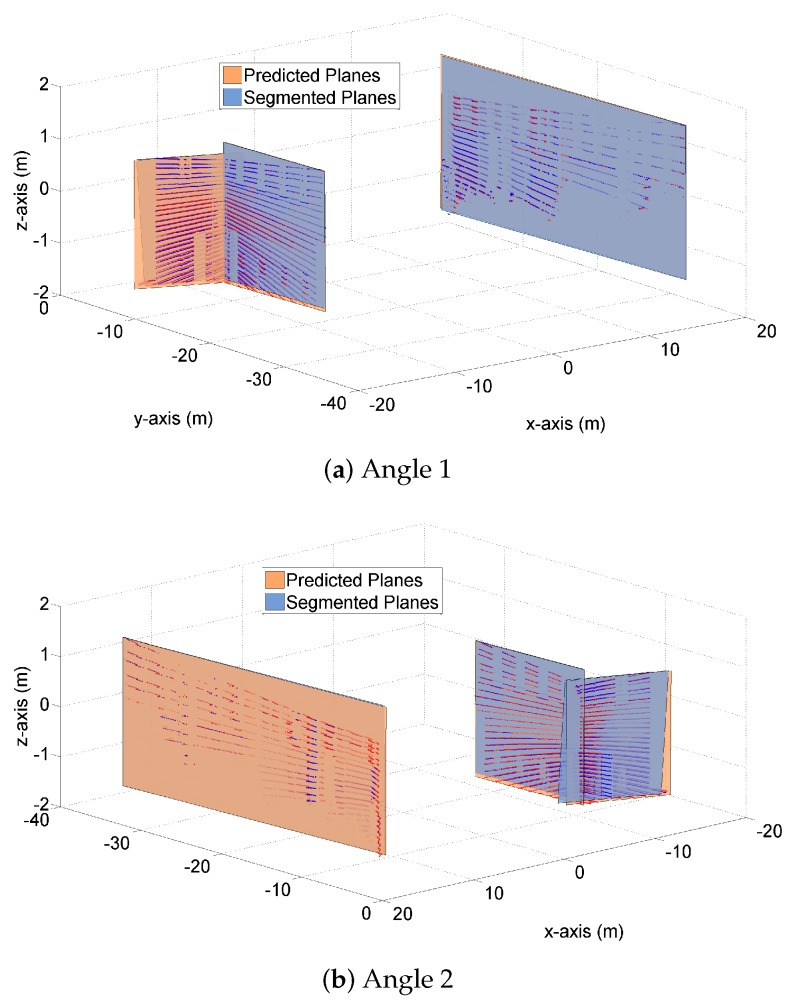

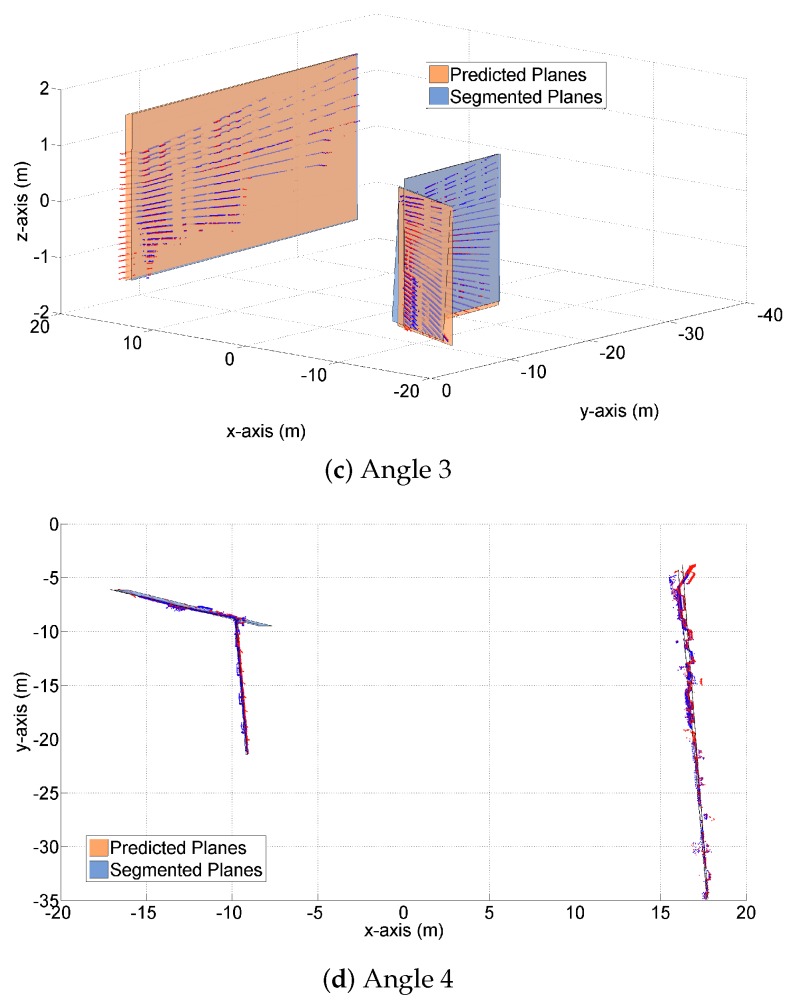
Multi-angle comparison of the predicted and segmented planes at time k=12.

In addition to k=125, our simulation studies have included a large range of other time steps. Figure 5 and Figure 6 provide the results of another time step k=12. The rest of time steps present similar results and are not shown in this paper for the purpose of brevity. In the following section, the above graphical results will be further supplemented and clarified, by means of detailed numerical results.

### 5.2. Numerical Results

In Figure 3, Figure 4, Figure 5 and Figure 6, we have graphically demonstrated the comparison results between the predicted and segmented planes, in terms of three important indicators – plane area, vertex distance and normal vector. In this section, we provide the results of our numerical simulation for 100 MC runs, in order to demonstrate ‘how close’ exactly these planes are.

The first two indicators, plane area and vertex distance, are mainly used to quantify the closeness in terms of plane dimensions (sizes). Table 1 shows the areas of the predicted and segmented planes for time step k=125 (averaged from 100 MC runs), and Table 2 gives the distances between each pair of plane vertexes for time step k=125 (averaged from 100 MC runs). We see in these tables that the areas of the predicted and segmented planes are close, and the distances between each pair of vertexes are small. These numerical results are consistent with the graphical results shown in Section 5.1.

The third indicator, normal vector, is employed to evaluate the closeness in terms of plane orientation. Table 3 shows the angles between each pair of normal vectors for time step k=125 (averaged from 100 MC runs) (‘Each pair of normal vectors’ is referred to the normal vector on the predicted plane and its counterpart on the segmented plane). The small magnitudes of these angles imply that the predicted and segmented planes are very close in terms of orientation. This explains the coincidence of the predicted and segmented planes seen in Figure 4c,d.

Besides the above three indicators for closeness evaluation, the execution time of the program is also recorded. The proposed transition model is implemented based on Algorithm 1, and the program is executed on a laptop equipped with an Intel i7-5600U CPU and an 8G RAM. For time step k=125, the execution time is 0.0375 s (averaged from 100 MC runs).

Apart from k=125, the numerical results for another time step k=12 are also presented in Table 4, Table 5 and Table 6. Similarly, these results show that the predicted planes (obtained using the information at k=11) are fairly close to the segmented planes at k=12, in terms of plane areas, vertex distances and normal vectors. Besides, the execution time of the program for k=12 is 0.0369 s (averaged from 100 MC runs). For the purpose of brevity, the numerical results for other time steps are omitted in this paper.

## 6. Conclusions

The majority of the current statistical SLAM solutions are based on using point features such as the representation of landmarks. The fast advancement of sensory technology makes it possible to utilize more sophisticated features in SLAM applications to achieve superior results while lowering computational cost. This paper presented the idea of using planar features for statistical SLAM, and proposed a stochastic transition model to propagate the plane parameters to the next time. A large range of simulation studies using real-world measurements have been conducted to evaluate the proposed transition model. Both graphical and numerical results show that the predicted planes generated by the proposed transition model closely match the segmented planes resulting from real-world point cloud measurements, in terms of three important indicators; plane area, vertex distance and normal vector. In the next step, we will look into other planar-feature-based stochastic transition models, and investigate the application of such transition models in statistical SLAM tasks.

## Figures and Tables

**Table 1:** Areas of the predicted and segmented planes for time step k=125.

	Plane Pair 1	Plane Pair 2	Plane Pair 3
Predicted Plane	$13.7147\;m^{2}$	$219.9937\;m^{2}$	$22.9218\;m^{2}$
Segmented Plane	$11.0470\;m^{2}$	$226.3712\;m^{2}$	$23.2926\;m^{2}$

**Table 2:** Distances between each pair of plane vertexes for time step k=125.

	Plane Pair 1	Plane Pair 2	Plane Pair 3
Vertex Pair 1	0.2503m	0.7089m	0.0603m
Vertex Pair 2	0.8519m	0.8667m	0.0294m
Vertex Pair 3	0.1293m	0.9060m	0.0837m
Vertex Pair 4	0.8107m	1.0730m	0.0677m

**Table 3:** Angles between each pair of normal vectors for time step k=125.

	Plane Pair 1	Plane Pair 2	Plane Pair 3
Angle Between Normal Vectors	0.0654rad/$3.7458^{\circ }$	0.0129rad/$0.7382^{\circ }$	0.0037rad/$0.2115^{\circ }$

**Table 4:** Areas of the predicted and segmented planes for time step k=12.

	Plane Pair 1	Plane Pair 2	Plane Pair 3
Predicted Plane	$34.5121\;m^{2}$	$22.7771\;m^{2}$	$91.5893\;m^{2}$
Segmented Plane	$33.7797\;m^{2}$	$23.0555\;m^{2}$	$90.5685\;m^{2}$

**Table 5:** Distances between each pair of plane vertexes for time step k=12.

	Plane Pair 1	Plane Pair 2	Plane Pair 3
Vertex Pair 1	0.1311m	0.8031m	0.1053m
Vertex Pair 2	0.1117m	0.1600m	0.1119m
Vertex Pair 3	0.0602m	0.2778m	0.5599m
Vertex Pair 4	0.0924m	1.0056m	0.5558m

**Table 6:** Angles between each pair of normal vectors for time step k=12.

	Plane Pair 1	Plane Pair 2	Plane Pair 3
Angle Between Normal Vectors	0.0034rad/$0.1936^{\circ }$	0.1133rad/$6.4912^{\circ }$	0.0070rad/$0.3987^{\circ }$

## Abbreviations

The following abbreviations are used in this manuscript:

SLAM	Simultaneous Localization and Mapping
EKF	Extended Kalman Filter
RFS	Random Finite Set
LMB	Labeled Multi-Bernoulli
CT	Constant Turn
ECT	Extended Constant Turn
SMC	Sequential Monte Carlo
EAP	Expected A Posteriori
MAP	Maximum A Posteriori
MSSE	Modified Selective Statistical Estimator
MC	Monte Carlo
